# Discrimination of Methionine Sulfoxide and Sulfone by Human Neutrophil Elastase

**DOI:** 10.3390/molecules26175344

**Published:** 2021-09-02

**Authors:** Darren Leahy, Cameron Grant, Alex Jackson, Alex Duff, Nicholas Tardiota, Jessica Van Haeften, Xingchen Chen, Jonathan M. Peake, Michael D. Kruppa, Eliot T. Smith, David A. Johnson, William B. Lott, Jonathan M. Harris

**Affiliations:** 1 School of Biomedical Science, Faculty of Health, Queensland University of Technology, Brisbane, QLD 4059, Australia; d2.leahy@qut.edu.au (D.L.); c23.grant@hdr.qut.edu.au (C.G.); alex.jackson@hdr.qut.edu.au (A.J.); alex.duff@hdr.qut.edu.au (A.D.); n.tardiota@qut.edu.au (N.T.); j.vanhaeften@hdr.qut.edu.au (J.V.H.); jonathan.peake@qut.edu.au (J.M.P.); william.lott@qut.edu.au (W.B.L.); 2 Centre for Excellence in Molecular Cell Science, Chinese Academy of Sciences, Shanghai 200031, China; xingchen.chen@connect.qut.edu.au; 3 Department of Biomedical Sciences, James H. Quillen College of Medicine, East Tennessee State University, Johnson City, TN 37614, USA; kruppa@etsu.edu (M.D.K.); DAVIDJ@etsu.edu (D.A.J.); 4 Department of Otolaryngology Head and Neck Surgery, Stanford School of Medicine, Stanford University, Stanford, CA 94305-5739, USA; eliotsmi@stanford.edu

**Keywords:** human neutrophil elastase, peptide aldehyde, substrate guided inhibitor design, methionine oxidation, methionine sulfone, substrate selectivity

## Abstract

Human neutrophil elastase (HNE) is a uniquely destructive serine protease with the ability to unleash a wave of proteolytic activity by destroying the inhibitors of other proteases. Although this phenomenon forms an important part of the innate immune response to invading pathogens, it is responsible for the collateral host tissue damage observed in chronic conditions such as chronic obstructive pulmonary disease (COPD), and in more acute disorders such as the lung injuries associated with COVID-19 infection. Previously, a combinatorially selected activity-based probe revealed an unexpected substrate preference for oxidised methionine, which suggests a link to oxidative pathogen clearance by neutrophils. Here we use oxidised model substrates and inhibitors to confirm this observation and to show that neutrophil elastase is specifically selective for the di-oxygenated methionine sulfone rather than the mono-oxygenated methionine sulfoxide. We also posit a critical role for ordered solvent in the mechanism of HNE discrimination between the two oxidised forms methionine residue. Preference for the sulfone form of oxidised methionine is especially significant. While both host and pathogens have the ability to reduce methionine sulfoxide back to methionine, a biological pathway to reduce methionine sulfone is not known. Taken together, these data suggest that the oxidative activity of neutrophils may create rapidly cleaved elastase “super substrates” that directly damage tissue, while initiating a cycle of neutrophil oxidation that increases elastase tissue damage and further neutrophil recruitment.

## 1. Introduction

Neutrophils of the human innate immune system use an armamentarium of chemical and enzymatic weapons to defend against pathogen invasion. Once triggered by pathogens, neutrophils respond with a shower of powerful oxidising agents including H_2_O_2_ [1]; superoxide [2]; hypochlorous and hypobromous acids [3] (through the action of myeloperoxidase) and release powerful proteolytic and lipolytic enzymes. The serine protease Human Neutrophil Elastase (HNE) is a released enzyme with both direct and indirect roles in bacterial killing by neutrophils [4,5]. However, HNE is also a causal factor in the progression of emphysema and chronic obstructive pulmonary disease (COPD) [6,7] due to the enzyme’s propensity to propagate cyclic inflammation, recruit neutrophils, and proteolytically destroy elastic lung tissue [8,9].

The degradation of inhibitors of other proteases makes HNE a uniquely destructive enzyme [10]. When HNE is dysregulated, the resulting protease-antiprotease imbalance initiates a proteolytic cascade that causes the characteristic pathologies observed in chronic respiratory diseases and other chronic conditions. To ameliorate the hazards of unrestrained HNE activity, the enzyme is targeted by reversible inhibitors, including the secretory leukoprotease inhibitor (SLPI) [11], elafin [12], and the irreversible serpin inhibitor alpha 1 anti-trypsin (A1AT), which predominately controls HNE activity in the lung. However, the potency of A1AT inhibition is entirely abrogated under oxidative conditions, such as those induced by a neutrophil response to infection, due to the oxidation of a methionine residue in the P1 position (Schechter–Berger nomenclature [13]) of its protease binding surface [14,15]. Although not completely abolished, the potencies of SLPI and elafin are diminished under oxidative conditions, due to the similar presence of methionine residues in their protease binding loops [16,17]. Oxidation of inhibitor methionine residues may be induced by oxidative stressors, such as cigarette smoke [18] or the respiratory burst of neutrophils [19], potentiating runaway proteolysis. Since these endogenous inhibitors bind to HNE in a substrate-like manner, the effects of methionine residue oxidation within peptide substrates might be expected to be similar to the inhibitors. However, the oxidation of P3 methionine residues in peptide substrates appears to enhance the HNE catalysed cleavage rates of those substrates [20] and suggests a strong positional dependence.

Seminal to this work, Kasperkiewicz et al. [20] screened a combinatorial library of unnatural (not genomically encoded) and natural amino acids to identify and develop an activity-based probe capable of distinguishing between HNE and the closely related protease neutrophil proteinase 3 (PR3). This library screen and analyses showed an unexpected HNE selectivity for an oxidised methionine residue at the P3 position of peptide substrates. Subsequent crystallography to characterise the interaction between the screen-selected activity-based probe PK101 and the HNE binding surface revealed an essential hydrogen bond between a sulfonyl oxygen of the oxidised methionine residue and the backbone amide of a unique Gly-Gly motif in the HNE S3 pocket [21]. Based on the interaction between PK101 and HNE, the authors posited that HNE adapted to incorporate the Gly-Gly motif to proteolytically cleave substrates containing oxidised P3 methionine residues. This notion is consistent with the observation of sulfone moieties in inhibitor drugs targeting HNE, including Sivelestat [22] and the Bayer inhibitor, BAY 85-8501 [23]. Thus, HNE activity enhancement by methionine oxidation at the P3 position of HNE substrates may hold special biological significance in vivo. However, PK101 and the optimal HNE peptide substrates selected from the combinatorial library contain unnatural amino acid residues [20], and the inhibitor drugs targeting HNE are small non-proteinaceous synthetic molecules [22,23]. Although the data strongly suggest that a P3 methionine sulfone residue in HNE substrates has biological relevance, a link has yet to be directly established. To better evaluate the biological relevance of HNE enhancement by the oxidised P3 methionine residue in peptide substrates, and to further clarify the reported HNE substrate interactions, we finely mapped HNE active site interactions using peptide substrates and inhibitors comprised of naturally occurring (genomically encoded) amino acids that contain a P3 methionine residue in the three available oxidation states: methionine, methionine sulfoxide and methionine sulfone.

## 2. Results

### 2.1. HNE Strongly Preferred Oxidised Methionine in Position P3 for Tetrapeptide pNA Substrates and Additional Positional Effects

**Figure 1:**
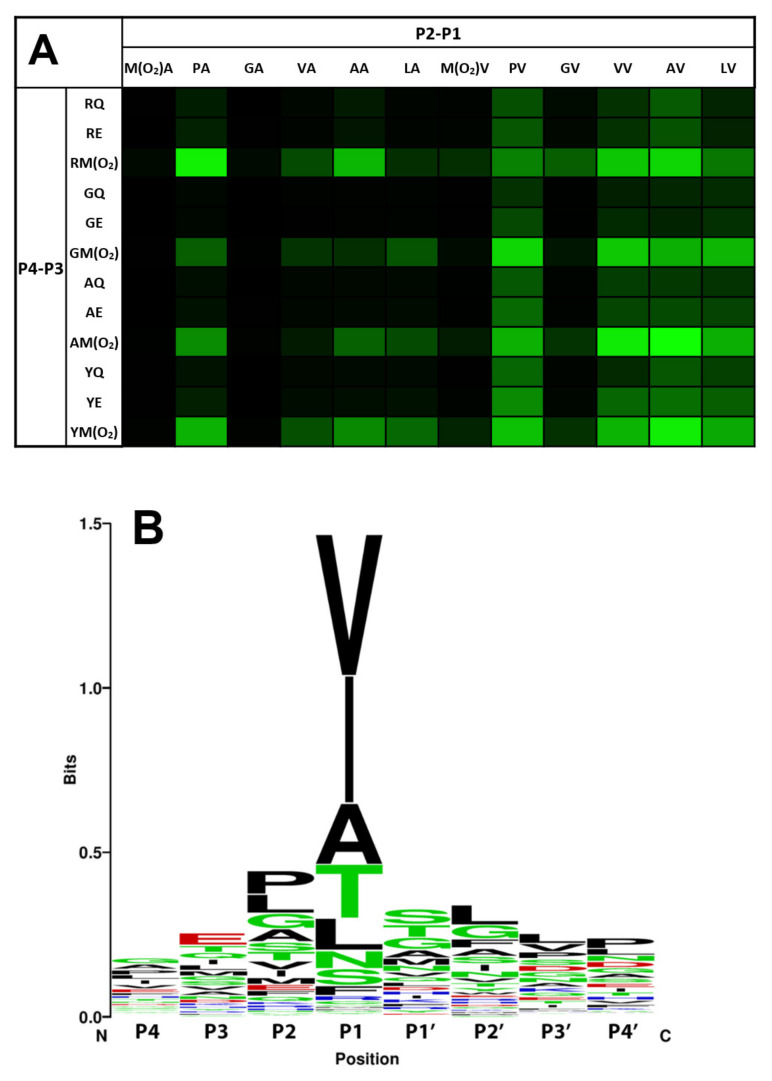
HNE substrate selectivity (**A**) heat map representation of tetra-peptide-pNA sparse matrix library screened for activity in HNE kinetic assays. Bright green cells represent peptides that showed high rates of HNE proteolysis, black cells represent peptides that showed no detectable HNE proteolysis. Each square represents the average rate from three replicate experiments of triplicate measurements. Final assay concentrations were 300 µM peptide-pNA and 1 µg/mL HNE. (**B**) HNE consensus sequence derived from the MEROPS database showing amino acid frequency distribution between positions P4 and P4′. Amino acids are coloured according to sidechain chemistry: black = non-polar; green = polar; red = negatively charged and blue = positively charged.

A focused sparse matrix library of tetrapeptides terminating in a para-nitroanilide (pNA) reporter group was assembled to probe the substrate selectivity of HNE. The library was constructed non-combinatorially as previously described [24], and the library design was guided by previous HNE selectivity studies [20,25,26,27]. HNE catalytic rates (mOD/min) were determined for each substrate, and the combined data are presented as a heat map in Figure 1A. Overall, selectivity for these individually synthesised peptides roughly follows the HNE consensus cleavage sequence found in the MEROPS database [28] shown in Figure 1B. These data confirm a strong selectivity for methionine sulfone residues at position P3, as peptide substrates that included a methionine sulfone residue at position P3 exhibited 19 of the 20 highest observed rates. This selectivity was position dependent, as peptides containing methionine sulfone in the P2 position made universally poor substrates. Interestingly, though unrelated to methionine oxidation, peptides containing glycine in the P2 position also made universally poor substrates.

### 2.2. Positional Effects and the Effect of N-Terminal Acetylation

**Figure 2:**
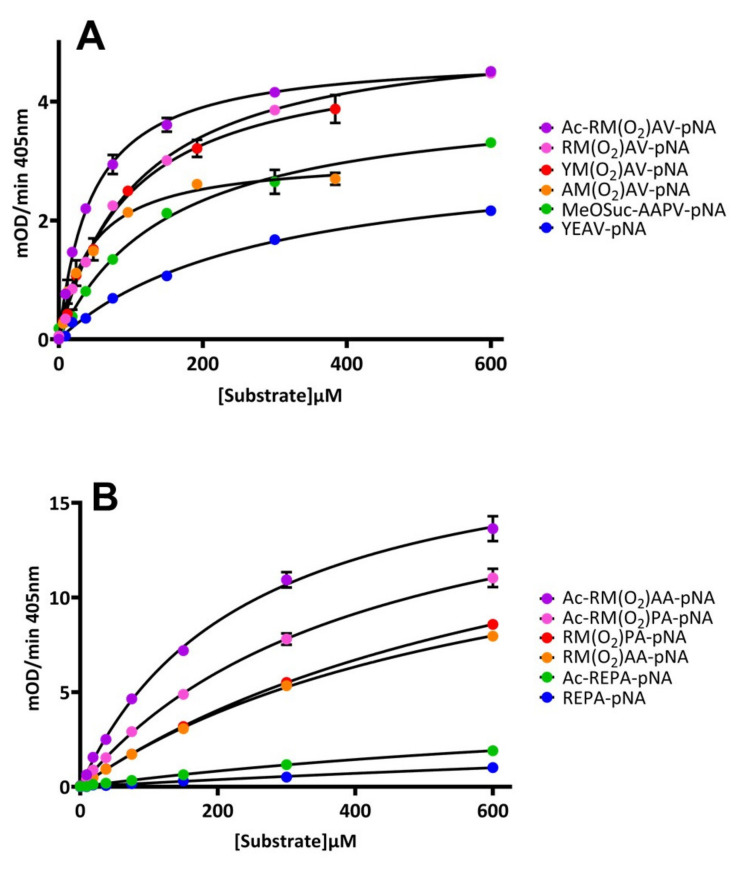
Substrate concentration versus velocity plot of candidate pNA substrates containing a P1 valine residue (**A**), or a P1 alanine residue (**B**), assayed against 3 nM active HNE. Data series were split between two figures due to the generally reduced maximum velocity of P1 valine substrates. Data points represent the average rate observed from three replicate experiments of triplicate measurements. Concentration ranges of YM(O_2_)AV-pNA and AM(O_2_)AV-pNA were reduced due to limiting solubility of these peptides. Error bars show SEM. Where not visible, error bars are smaller than the pictured data point.

To better understand the interactions of the peptide substrates with HNE, five substrates among those exhibiting the highest catalytic rates were chosen for synthesis and further analysis, along with two substrates showing intermediate and low catalytic rates. The standard substrate MeOSuc-AAPV-pNA was also synthesised to provide a benchmark against other studies. P1 alanine residues showed the highest catalytic rates (k_cat_), but P1 valine residues showed the strongest substrate affinity. Acetylated forms of the P4 arginine substrates were synthesised to assess the effect of a charged N-terminus. Acetylation had little effect on k_cat_, but reduced a given substrate’s K_M_ by approximately one-half (Figure 2 and Table 1). The K_M_ for MeOSuc-AAPV-pNA was found to be 0.152 mM compared to a previously published value of 0.14 mM [29].

### 2.3. A Peptide Aldehyde Inhibitor of HNE as a Probe of HNE Substrate Interactions

Given the strong positional effects apparent for HNE, peptide aldehydes were used as reversible transition state analogue inhibitors to further investigate the role of the peptide sequence in HNE interactions. Since these inhibitors bind at serine protease active sites similarly to their cognate substrates, peptide aldehyde inhibition is an appropriate proxy for quantitating peptide/protease interactions. The most efficient HNE peptide substrate, Ac-RM(O_2_)AV-pNA, was synthesised as its peptide aldehyde equivalent, Ac-RM(O_2_)AV-H, and characterised in an inhibition assay. The inhibition constant (K_i_) for the Ac-RM(O_2_)AV-H inhibition of HNE proteolysis was determined to be 147 nM (Table 2).

### 2.4. Peptide Aldehyde Inhibitors Show Differential Potency Dependent on P3 Methionine Oxidation Status

**Figure 3:**
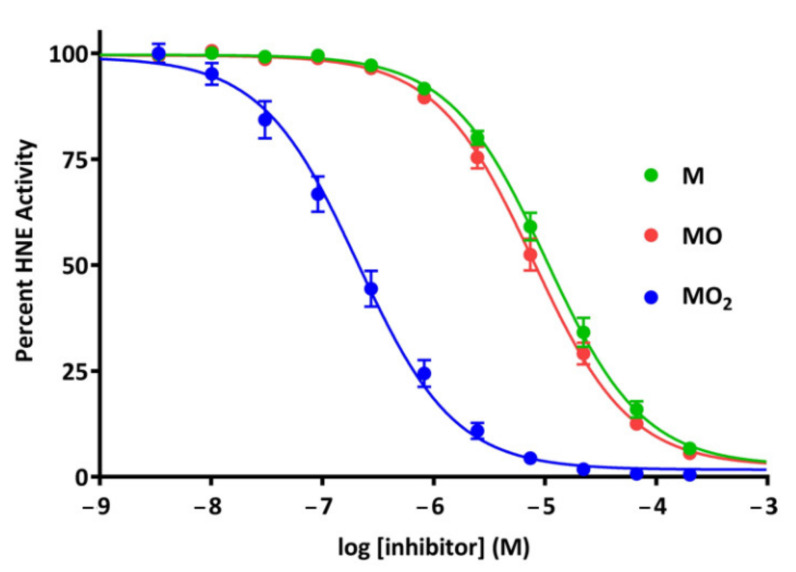
Dose-response plot of Ac-RMAV-H peptide aldehyde inhibitor variants containing differing oxidation states of the P3 methionine residue. All assays contained 10 nM active HNE and 100 nM Ac-RM(O_2_)AA-pNA substrate. Data are reported as percent activity remaining compared to uninhibited controls, and data points represent the mean observed from three replicate experiments of triplicate measurements. Error bars show SEM. Where not visible, error bars are smaller than the pictured data point.

The incorporation of pNA as a reporter group necessitated oxidation of the peptide substrates during synthesis. Thus, synthesis of pNA substrates containing non-oxidised methionine residues was not feasible. However, peptide aldehydes can be synthesised to incorporate methionine residues in each of the available methionine oxidation states. These inhibitors can effectively assess the interactions of methionine, methionine sulfoxide and methionine sulfone with HNE using catalytic inhibition as a readout. The sulfone derivative Ac-RM(O_2_)AV-H exhibited a 49.4-fold inhibition enhancement relative to the non-oxidised Ac-RMAV-H. By contrast, the sulfoxide derivative Ac-RM(O)AV-H enhanced inhibition by only 1.3-fold compared to Ac-RMAV-H (Figure 3 and Table 2).

### 2.5. Molecular Modelling Suggests a Role for a Discrete Solvent Interaction in Binding Selectivity between Methionine Residue Oxidative Forms

**Figure 4:**
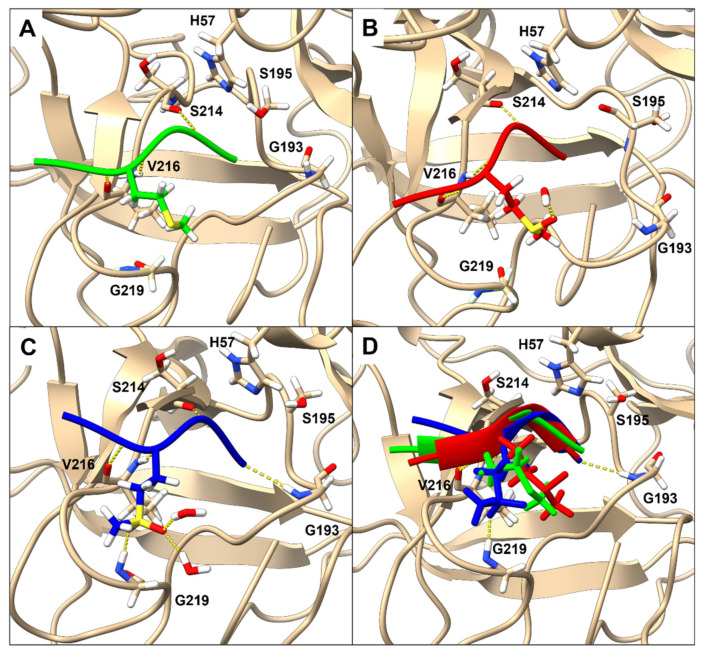
HNE in complex with peptide aldehyde inhibitors (**A**) Ac-RMAV-H, (**B**) Ac-RM(O)AV-H, (**C**) Ac-RM(O_2_)AV-H, and (**D**) overlayed. Each complex used the final snapshot from a 10 ns simulation and was energy minimised prior to alignment of the backbone atoms. Each peptide is represented by a ribbon backbone structure and key P3 methionine sidechain that illustrates their modified binding pattern to HNE. Measurement of the distance between carbonyl atoms for the three variants revealed a 1.52 Å disparity between methionine sulfone versus native methionine, and only a 0.18 Å displacement between methionine sulfoxide and native methionine. This shift in conjunction with the hydrogen bond seen between Gly218/219 would suggest that the movement of the peptide backbone is caused by the hydrogen bond network of this loop.

To investigate the role performed by methionine oxidation in HNE/peptide interactions, molecular dynamic simulations for each methionine residue oxidation state of the aldehyde inhibitor with HNE in the presence of an explicit solvent was obtained. Predicted hydrogen bonding interactions and sidechain dispositions for each of the inhibitors are shown in Figure 4. The model predicts that the side chain of the P3 methionine and P3 methionine sulfoxide residues will deflect away from the active site, whilst the P3 methionine sulfone residue side chain will insert into the shallow HNE S3 pocket aided by a partial occupancy (20%) hydrogen bond between the second sulfonyl oxygen and the main chain of Gly193. Hydrogen bond interactions are predicted between discrete water molecules and either the sulfinyl oxygen of the methionine sulfoxide residue or one of the sulfonyl oxygens of the methionine sulfone residue.

## 3. Discussion

### 3.1. Tetrapeptide Substrates Composed of Naturally Occurring Amino Acids Containing a P3 Methionine Sulfone Residue Enhance HNE Catalysis

The data reported here are consistent with a significant biological role for the previously reported [20] HNE enhancement by substrates containing a P3 methionine sulfone residue. The highest HNE catalysed rates were observed for peptide substrates from our library containing a P3 methionine sulfone residue. Further, a peptide aldehyde based on a preferred peptide substrate sequence inhibited HNE with a K_i_ of 147 nM. As our library contained only peptide substrates comprised of genomically encoded amino acids, this suggests that HNE can specifically target naturally occurring substrates in vivo under neutrophil-induced oxidative conditions. The observations that either methionine sulfone residues in the P2 position or glycine in the P2 position yielded universally poor HNE substrates, confirm a positional dependence dictated by the bonding surface.

### 3.2. HNE Substrate Selectivity and Positional Effects

Oxidation of methionine is a two-step process, in which the methionine residue is first oxidised to the sulfoxide and then irreversibly oxidised to the sulfone [30]. Taken together, the data reported here for the peptide substrates and the peptide aldehyde-based inhibitors suggest that HNE discriminates between the sulfoxide and sulfone forms of oxidised P3 methionine residue in the S3 pocket. The observation that the sulfone derivative Ac-RM(O_2_)AV-H exhibited a 49.4-fold inhibition enhancement relative to the non-oxidised Ac-RMAV-H suggests that the inhibition enhancement largely stemmed from increased inhibitor binding. Thus, the likely effect of P3 methionine oxidation to the sulfone in HNE substrates is to lower the K_m_.

A pronounced HNE preference for peptide substrates with valine at P1 was previously reported [20]. In our hands, the P1 alanine substrates among the five “high rate” substrates chosen from our screen exhibited greater HNE catalytic turnover (k_cat_) than the P1 valine substrates by at least a factor of 3.5 (Table 1). However, when catalytic efficiency (k_cat_/K_m_) was analysed, no clear distinction between P1 alanine and P1 valine emerged. With respect to P1 valine efficiency, strong substrate affinity offset weak catalysis in the binding site, while with respect to P1 alanine efficiency, poor binding affinity was offset by strong catalysis. The difference in P1 preference observed between library screens is likely an artefact of the different reaction conditions used. Library screening results using protocols based on catalytic rate as a readout differ depending on whether the protocol was performed under enzyme saturating conditions. Under sub-saturating conditions, the rate expression is $v=\frac{kcat}{Km}$ [E][S]. Sub-saturated screening favours substrates with smaller K_m_s (i.e., higher binding affinity) to compensate when [S] is very small. Under saturating conditions, the rate expression simplifies to $v=kcat\left[E\right]$, and screening favours substrates with faster rates of catalysis in the enzyme binding site (k_cat_), without regard to substrate binding affinity (K_m_). The previous combinatorial library [20] was constructed of pools of many thousands of peptides, each in very low concentration, and each individual substrate concentration was likely to be far below its respective K_m_. Thus, screening selection was likely dominated by substrate binding affinity (K_m_). By contrast, our sparse matrix library consisted of only 144 total substrates, each substrate present at a concentration of 300 µM. Excluding REPA-pNA, which was chosen as an example of a poor HNE substrate, only two of the remaining six chosen pNA substrates listed in Table 1 had K_m_ values greater than 300 µM. Those two, RM(O2)PA-pNA and RM(O2)AA-pNA, had K_m_ values that were only approximately twice the substrate concentration of the screen, well-within an order of magnitude. Therefore, our library screen selection was primarily dominated by k_cat_. This might explain why the combinatorial library screening would select strong-binding substrates incorporating unnatural amino acid residues over weaker-binding substrates composed only of natural amino acid residues, which might have greater biological relevance. This sub-saturating characteristic might be useful for strong-binding substrate applications, such as identifying competitive inhibitor candidates from a combinatorial library screen.

Finally, unlike the tetrapeptide substrates used in the sparse matrix library, biologically relevant substrate cleavage sites are embedded in the interior of longer protein chains, and therefore rarely near the positively charged N-terminus. To mimic this condition, we acetylated the best tetrapeptide substrates chosen in our library screen to produce uncharged N-termini. N-terminal acetylation increased substrate binding affinity to HNE without appreciably affecting the catalysis mechanism in the active site.

### 3.3. The Role of Bulk Water in the HNE Mechanism of Discrimination against P3 Methionine Sulfoxide

The crystal structure of the methionine sulfonyl-containing inhibitor PK-101 [21] identified a hydrogen bond between the oxidised methionine residue present in PK-101 and the main chain amide at Gly219 in the S3 pocket of HNE. The associated diglycine motif is unique to HNE among all human serine proteases and is a hallmark of an adaptation to oxidised substrates. Our kinetic data and molecular modelling predictions are consistent with an important role for P3 methionine sulfone substrates in HNE proteolytic activity enhancement via a mechanism similar to that described for PK-101 [21]. The P3 methionine sulfoxide residue, which is a far more common oxidative product in vivo than the sulfone, surprisingly failed to enhance interactions with HNE despite containing a sulfinyl oxygen similarly positioned to a hydrogen bond in the HNE S3 pocket. Indeed, molecular dynamic simulations predict that the methionine sulfoxide sidechain will deflect away from the S3 pocket and be solvent exposed. Closer examination of the molecular dynamics trajectories revealed solvent hydrogen bonding with the sulfinyl and sulfone oxygens of the P3 methionine sulfoxide residue and P3 methionine sulfone residue. However, as secondary amides are significantly weaker hydrogen bond donors than water [31], the backbone amide hydrogen bond donor at Gly219 is thermodynamically unable to compete with the solvent water molecule for the single sulfinyl oxygen hydrogen bond acceptor on the methionine sulfoxide residue. In contrast, the methionine sulfone residue contains two sulfonyl oxygens, and is predicted to simultaneously form hydrogen bonds with both the solvent water molecule and the main chain amide at Gly219 (see Figure S1 for trajectories). As our aldehyde HNE inhibitors suggest that the P3 methionine sulfone residue increases binding affinity, decreasing the K_m_ would explain why a P3 methionine sulfone enhances HNE catalysis while a P3 methionine sulfoxide does not.

### 3.4. Biological and Clinical Implications of Selection for P3 Methionine Sulfone

Whilst the mechanism of selection between the two forms of oxidised methionine is clear, the biological justification for this discrimination by HNE seems more obscure. Although unusual, oxidised methionine residues have been observed in biological samples [32]. Why would it be important to cleave one form of oxidised methionine but not the other? The biological significance may lie in the way that methionine sulfoxide and methionine sulfone residues are repaired by pathogens. The sulfoxide moiety is a substrate for repair enzymes that are vital for bacterial resistance to neutrophil killing. These enzymes, methionine sulfoxide reductase A and B, convert partially oxidised methionine back to the reduced form. Significantly, methionine sulfoxide reductase knockout bacteria are sensitized to the cell killing effects of hydrogen peroxide [33,34]. Hence, bacteria can readily repair the oxidative “cut here” tags affixed by neutrophil activity so long as oxidation does not progress to the sulfone state.

The potential clinical consequences of HNE’s methionine sulfone selectivity are two-fold. Firstly, the neutrophil oxidative burst has the capacity to target HNE activity towards pathogen-derived proteins that were oxidised, and secondly, host proteins that are collaterally exposed to oxidation might also become preferred substrates. Accelerated cleavage of pathogen proteins, especially virulence factors, would be advantageous, as it would represent a secondary targeted attack by HNE on the invading pathogens. Examples of HNE pathogen substrates include outer membrane protein A (OmpA) in *E. coli*, where its degradation is lethal [35], and the cleavage of flagellin from *Pseudomonas aeruginosa*, which abrogates its biological activity [36]. Both of these targets have consensus cleavage sequences defined in the MEROPS database for HNE that contain oxidisable methionine residues (Flagellin E_163_MSA, OmpA G_141_MLS) [28]. In contrast to the beneficial effects of accelerated pathogen degradation, oxidation of host proteins and their accelerated cleavage by HNE would lead to increased rates of collateral tissue damage. This in turn would have the potential to set up a chronic cycle of oxidation followed by tissue damage and further recruitment of neutrophils. Such collateral damage may also be occurring in acute conditions such as the lung damage caused by COVID-19 infection.

### 3.5. Conclusions

In the pursuit of understanding how methionine oxidation modulates substrate and inhibitor interactions with human neutrophil elastase, there is one critical question that must be asked: is the modification of P3 methionine to methionine sulfone able to be induced by natural mechanisms, or is it only applicable to synthetic molecules and unnatural amino acid residues? The Gly-Gly motif that is unique to HNE suggests an evolutionary pressure to conserve the observed methionine sulfone selectivity in HNE, and a mouse model of radiation-induced oxidative stress [37] shows that it is certainly possible for methionine residues to oxidise to the sulfone in vivo. However, methionine oxidation to the sulfone is infrequent, and depends on a combination of strong oxidative stress and the intrinsic vulnerability of only select methionine residues. Using tetrapeptide substrates composed entirely of naturally occurring amino acids, we showed that P3 methionine residue oxidation to the sulfone may be biologically relevant, but this remains to be demonstrated in biologically relevant protein molecules.

We showed that oxidation of methionine to the sulfone in HNE-interacting peptides greatly increases their affinity for the enzyme. This and the demonstrated ability of endogenous oxidation systems to inactivate HNE’s chief inhibitor, may justify research that investigates the utility of antioxidant treatment in acute and non-acute immune conditions such as COVID-related respiratory distress and COPD.

## 4. Materials and Methods

### 4.1. Peptide Synthesis

To produce the 144 tetra-peptide pNA substrates we employed standard Fmoc solid phase peptide synthesis (SPPS) protocols [38]. The scale of the library synthesis was such that 0.05 mmol of each final peptide was to be produced, a total of 7.20 mmol. Into large Bio-Rad Poly-Prep columns, 4.8 g of 2-chlorotrityl resin (1.52 mmol available Cl/g) was derivatised with an 8x molar equivalent of both para-phenylenediamine (pPD) and diisopropylethylamine (DIPEA) and solubilised in dimethylformamide (DMF) to bring final the DIPEA concentration to 10%. Columns were covered with aluminium foil to exclude light and left slowly rotating for 16 h at RT. After pPD derivatisation, the resin was drained, then washed with DMF three times to remove all excess reactants. Unoccupied 2-chorotrityl groups were capped by treatment with a column volume of 10% methanol, slowly rotated for 30 min at RT, drained and washed with DMF three times. After pPD derivatization chain elongation followed conventional practice with 50% piperidine in DMF as deprotectant and coupling effected by activating Fmoc amino acids with 1.1 Meq of 2-(6-Chloro-1H-benzotriazole-1-yl)-1,1,3,3-tetramethylaminium hexafluorophosphate (HCTU). The resin was washed three times with vacuum aspiration between each step. Both coupling and deprotection were agitated by sparging with dried nitrogen. Once chain elongation was completed, peptides were cleaved from the 2-chlorotrityl resin by treatment with 5% Trifluoroacetic acid (TFA) in DCM keeping side chain protection intact and harvested by trituration in 10 volumes of ice-cold diethyl ether. The para-aminoanilide group of the harvested peptides was oxidised by treatment with 4 Meq of Oxone. Briefly, peptides were re-solubilised with acetonitrile:water (1:1 *v*/*v*) containing Oxone and left mixing overnight at RT protected from light. Ethyl acetate was used to extract the oxidised protected peptides from the aqueous oxidation solution and was subsequently removed by evaporation under a stream of dried compressed air. RMAV-H peptide aldehydes were synthesised using an oxazolidine protection approach on Novabiochem H-Thr-Gly-NovaSyn TG resin (Merck). This resin was purchased preloaded with Fmoc-valinal (0.17 mmol/g equivalent) at a density of 0.5 mM/g. Chain elongation was achieved as with the pNA substrates. Prior to oxazolidine cleavage, the amino acid side chains required deprotection by treatment with anhydrous TFA for 30 min and washing with 3 x DMF followed by 3 x DCM washes. Once completed, the peptide was released from the synthesis resin using a mixture of 10% acetic acid, 5% water, 63% DCM and 21% methanol prior to trituration as for the pNA substrates. RM(O)AV-H was synthesised using a building block approach incorporating Fmoc-Met(O) at P3 and RM(O_2_)AV-H was formed by subjecting RMAV-H to on-resin oxidation with 6 Meq of Oxone in 50% acetonitrile at the end of the chain elongation. Substrates for kinetic analysis and the aldehyde inhibitors were both subject to solid phase extraction on Grace C18 SPE columns particle size: 50 µm; column phase: reversed; bed weight: 900 mg, before purification on a Kinetex 5 μm XB-C18 100 Å RP-HPLC column (Phenomenex) with peptides eluted with a gradient of 10–100% acetonitrile in ultrapure water with 0.1% TFA as a modifier. Peptide elution was monitored at 214 nm. Post purification, peptide containing fractions were analysed by LC-MS on a Sciex QTrap 4500 Q1 scan over 50–1500 m/z. Mobile phase used was 40% acetonitrile containing 0.1% formic acid as a modifier. Validated peptides were subject to lyophilization, weighed and either redissolved in 30% isopropanol at a concentration of 6 mM and stored frozen at −20 °C, or for long term storage peptides were maintained as dried powders at −80 °C.

### 4.2. Enzyme Kinetic Assays

For substrate library kinetic assay screen, reactions were initiated by addition of HNE purified from purulent human sputum (Elastin Products Company, Owensville, MO, USA) to triplicate microplate wells containing each substrate, and the rate of reaction determined spectrophotometrically at 405 nm. Final concentrations were 300 µM substrates and 1 µg/mL HNE in 250 µL assay buffer (100 mM Tris pH 8, 50 mM NaCl, 10% DMF, 0.05% Triton X-100). For in-depth assays of candidate substrates and inhibitors, the active site concentration of HNE was first determined by titration with A1AT. Briefly, 250 nM A1AT was serially diluted in a 96-well microplate to provide a final assay volume of 250 µL. A total of 2.5 µL of HNE stock solution (100 µg/mL) was added to each well and complex formation was allowed to proceed over a period of 20 min at RT. Assays were initiated by addition of Ac-RM(O_2_)PA-pNA substrate, monitoring the evolution of free para-nitroanilide at 405 nm for 90 s again at RT. Kinetic assays of synthesized pNA substrates were carried out similarly omitting the A1AT and serially diluting the substrates, K_M_ and Vmax values were determined by GraphPad Prism 5.01 (La Jolla, CA, USA) Michaelis–Menten non-linear regression. Assays with peptide aldehyde inhibitors were carried out as with the active site titration. Inhibition constants were calculated in GraphPad Prism by non-linear regression using the Morrison equation for tight-binding inhibitors [39].

### 4.3. Molecular Modelling

The crystal structure 4WVP determined by Lechtenberg et al. [21] was used as the basis for predicting inhibitor/HNE interactions. Initially, the bound probe in 4WVP was converted to the natural amino acid containing peptides RMAV-H, RM(O)AV-H and RM(O_2_)AV-H within the YASARA suite of programs. Molecular simulation on each of the resulting complexes was carried out with VMD 1.9.2 [40] and NAMD 2.9 [41] packages on a GPU cluster built in-house. HNE/Inhibitor complexes were solvated in TIP3P water, in a simulation cell neutralised with 0.1 M Na^+^ and Cl^−^ counter ions and parameterised with the CHARMM27 force field [42]. Each of the three complexes were subject to molecular dynamics consisting of a three-step relaxation followed by three independent production runs of 10 ns simulation time [43]. In the first step, the integration time step was set to 1 fs and harmonic restraints of 2 kcal·mol^−1^·Å^−2^ was applied on all heavy atoms. A 1000-step conjugate gradient minimisation was performed followed by 500 ps of molecular dynamics simulation. In the second step, and restraints retained on protein Cα atoms and the system was equilibrated for 1 ns with 2 fs time step. In the third step, the system was simulated for 1 ns with all restraints removed. After equilibration, three independent production runs (20 ns each run) were performed in the NPT ensemble. In all simulations, Langevin dynamics were used to maintain constant temperature (298 K) with a damping coefficient of 5 ps^−1^. Pressure within the simulation cell was regulated with a Langevin piston barostat with an oscillation period of 100 fs maintaining a constant 1 atm. Long-range electrostatics were treated by the Particle-Mash-Ewald (PME) method with a 9 Å cut-off. Snapshots were saved every 1 ps. The analysis of simulation trajectories was performed on VMD 1.9.2 [40]. Hydrogen bond length and angle cut-offs were set to 3.3 Å and 40°.

## Figures and Tables

**Table 1:** Kinetic constants calculated for candidate HNE pNA substrates. Substrates in each section are sorted by descending k_cat_ values. K_M_ values are listed ± SEM.

	Substrate	k_cat_ (sec^−1^)	K_M_ (µM)	k_cat_/K_M_ (M^−1^sec^−1^)
**High Rate**	RM(O_2_)PA-pNA	13.72	765.1 ± 51.2	1.79 × 10^4^
	RM(O_2_)AA-pNA	11.60	641.5 ± 51.8	1.81 × 10^4^
	RM(O_2_)AV-pNA	3.70	107.7 ± 5.2	3.43 × 10^4^
	AM(O_2_)AV-pNA	2.16	43.7 ± 5.7	4.95 × 10^4^
	YM(O_2_)AV-pNA	3.42	96.8 ± 11.0	3.53 × 10^4^
**Intermediate Rate**	YEAV-pNA	2.24	280.0 ± 28.6	7.99 × 10^3^
**Low Rate**	REPA-pNA	4.22	2993 ± 1687	1.41 × 10^3^
**Acetylated**	Ac-RM(O_2_)AA-pNA	13.50	239.5 ± 20.4	5.64 × 10^4^
	Ac-RM(O_2_)PA-pNA	13.14	417.7 ± 39.5	3.15 × 10^4^
	Ac-REPA-pNA	3.82	1113 ± 360.9	3.44 × 10^3^
	Ac-RM(O_2_)AV-pNA	3.37	45.8 ± 2.3	7.36 × 10^4^
**Reference**	MeOSuc-AAPV-pNA	2.90	152.9 ± 17.6	1.89 × 10^4^

**Table 2:** Comparison of the HNE peptide aldehyde inhibitor Ac-RMAV-H with derivatives containing P3 methionine residues in sulfoxide and sulfone forms.

	K_i_ (nM)	Relative Potency
Ac-RMAV-H	7266	1
Ac-RM(O)AV-H	5547	1.3
Ac-RM(O_2_)AV-H	147	49.4

## Data Availability

Not available.

## Supplementary Materials

Figure S1. Trajectories for molecular dynamic simulation of HNE in complex with RMAV-H peptide aldehyde and its oxidized variants. Figure S2. SDS PAGE analysis of HNE supplied by Elastin Products Company. Table S1. Calculated and observed masses of candidate peptide-pNA substrates and peptide aldehyde inhibitors. All masses are listed in Daltons.

## Sample Availability

Samples of theparanitroanilide substrates used in this study are available from the authors.

## References

[1] Weiss S.J., Young J., LoBuglio A.F., Slivka A., Nimeh N.F. (1981). Role of hydrogen peroxide in neutrophil-mediated destruction of cultured endothelial cells. J. Clin. Investig..

[2] Babior B.M., Curnutte J.T., McMurrich B.J. (1976). The particulate superoxide-forming system from human neutrophils. Properties of the system and further evidence supporting its participation in the respiratory burst. J. Clin. Investig..

[3] Van Dalen J.C., Whitehouse W.M., Winterbourn C.C., Kettle J.A. (1997). Thiocyanate and chloride as competing substrates for myeloperoxidase. Biochem. J..

[4] Belaaouaj A., McCarthy R., Baumann M., Gao Z., Ley T.J., Abraham S.N., Shapiro S.D. (1998). Mice lacking neutrophil elastase reveal impaired host defense against gram negative bacterial sepsis. Nat. Med..

[5] Standish A.J., Weiser J.N. (2009). Human neutrophils kill Streptococcus pneumoniae via serine proteases. J. Immunol..

[6] Stockley R.A. (1999). Neutrophils and Protease/Antiprotease Imbalance. Am. J. Respir. Crit. Care Med..

[7] Tetley T.D. (1993). New perspectives on basic mechanisms in lung disease. 6. Proteinase imbalance: Its role in lung disease. Thorax.

[8] Snider G.L., Lucey E.C., Christensen T.G., Stone P.J., Calore J.D., Catanese A., Franzblau C. (1984). Emphysema and bronchial secretory cell metaplasia induced in hamsters by human neutrophil products. Am. Rev. Respir. Dis..

[9] Sandhaus R.A., Turino G. (2013). Neutrophil Elastase-Mediated Lung Disease. COPD J. Chronic Obstr. Pulm. Dis..

[10] Jackson P.L., Xu X., Wilson L., Weathington N.M., Clancy J.P., Blalock J.E., Gaggar A. (2010). Human Neutrophil Elastase-Mediated Cleavage Sites of MMP-9 and TIMP-1: Implications to Cystic Fibrosis Proteolytic Dysfunction. Mol. Med..

[11] Rice W., Weiss S. (1990). Regulation of proteolysis at the neutrophil-substrate interface by secretory leukoprotease inhibitor. Science.

[12] Wiedow O., Schröder J.M., Gregory H., Young J.A., Christophers E. (1990). Elafin: An elastase-specific inhibitor of human skin. Purification, characterization, and complete amino acid sequence. J. Biol. Chem..

[13] Schechter I., Berger A. (1967). On the size of the active site in proteases. I. Papain. Biochem. Biophys. Res. Commun..

[14] Johnson D., Travis J. (1979). The oxidative inactivation of human alpha-1-proteinase inhibitor. Further evidence for methionine at the reactive center. J. Biol. Chem..

[15] Taggart C., Cervantes-Laurean D., Kim G., McElvaney N.G., Wehr N., Moss J., Levine R.L. (2000). Oxidation of either Methionine 351 or Methionine 358 in α1-Antitrypsin Causes Loss of Anti-neutrophil Elastase Activity. J. Biol. Chem..

[16] Boudier C., Bieth J.G. (1994). Oxidized mucus proteinase inhibitor: A fairly potent neutrophil elastase inhibitor. Biochem. J..

[17] Nobar S.M., Zani M.-L., Boudier C., Moreau T., Bieth J.G. (2005). Oxidized elafin and trappin poorly inhibit the elastolytic activity of neutrophil elastase and proteinase 3. FEBS J..

[18] Biagi G.L., Fregnan G.B., Gazzani G., Vandoni G. (1989). Erdosteine protection from cigarette smoke-induced loss of alpha 1-antitrypsin activity in rat lungs. Int. J. Clin. Pharmacol. Ther. Toxicol..

[19] Hubbard R.C., Ogushi F., Fells G.A., Cantin A.M., Jallat S., Courtney M., Crystal R.G. (1987). Oxidants spontaneously released by alveolar macrophages of cigarette smokers can inactivate the active site of alpha 1-antitrypsin, rendering it ineffective as an inhibitor of neutrophil elastase. J. Clin. Investig..

[20] Kasperkiewicz P., Poreba M., Snipas S.J., Parker H., Winterbourn C.C., Salvesen G.S., Drag M. (2014). Design of ultrasensitive probes for human neutrophil elastase through hybrid combinatorial substrate library profiling. Proc. Natl. Acad. Sci. USA.

[21] Lechtenberg B.C., Kasperkiewicz P., Robinson H., Drag M., Riedl S.J. (2015). The Elastase-PK101 Structure: Mechanism of an Ultrasensitive Activity-based Probe Revealed. ACS Chem. Biol..

[22] Kawabata K., Suzuki M., Sugitani M., Imaki K., Toda M., Miyamoto T. (1991). ONO-5046, a novel inhibitor of human neutrophil elastase. Biochem. Biophys. Res. Commun..

[23] Von Nussbaum F., Li V.M., Allerheiligen S., Anlauf S., Bärfacker L., Bechem M., Delbeck M., Fitzgerald M.F., Gerisch M., Gielen-Haertwig H. (2015). Freezing the Bioactive Conformation to Boost Potency: The Identification of BAY 85-8501, a Selective and Potent Inhibitor of Human Neutrophil Elastase for Pulmonary Diseases. ChemMedChem.

[24] Swedberg J., Nigon L.V., Reid J.C., de Veer S., Walpole C.M., Stephens C.R., Walsh T.P., Takayama T.K., Hooper J., Clements J. (2009). Substrate-Guided Design of a Potent and Selective Kallikrein-Related Peptidase Inhibitor for Kallikrein 4. Chem. Biol..

[25] Harris J.L., Backes B.J., Leonetti F., Mahrus S., Ellman J.A., Craik C.S. (2000). Rapid and general profiling of protease specificity by using combinatorial fluorogenic substrate libraries. Proc. Natl. Acad. Sci. USA.

[26] Schilling O., Overall C.M. (2008). Proteome-derived, database-searchable peptide libraries for identifying protease cleavage sites. Nat. Biotechnol..

[27] Fu Z., Thorpe M., Akula S., Chahal G., Hellman L.T. (2018). Extended Cleavage Specificity of Human Neutrophil Elastase, Human Proteinase 3, and Their Distant Ortholog Clawed Frog PR3—Three Elastases With Similar Primary but Different Extended Specificities and Stability. Front. Immunol..

[28] Rawlings N.D., Barrett A.J., Thomas P., Huang X., Bateman A., Finn R.D. (2018). The MEROPS database of proteolytic enzymes, their substrates and inhibitors in 2017 and a comparison with peptidases in the PANTHER database. Nucleic Acids Res..

[29] Castillo M.J., Nakajima K., Zimmerman M., Powers J.C. (1979). Sensitive substrates for human leukocyte and porcine pancreatic elastase: A study of the merits of various chromophoric and fluorogenic leaving groups in assays for serine proteases. Anal. Biochem..

[30] Hoshi T., Heinemann S.H. (2001). Regulation of cell function by methionine oxidation and reduction. J. Physiol..

[31] Eberhardt E.S., Raines R. (1994). Amide-Amide and Amide-Water Hydrogen Bonds: Implications for Protein Folding and Stability. J. Am. Chem. Soc..

[32] Kim G., Weiss S.J., Levine R.L. (2014). Methionine oxidation and reduction in proteins. Biochim. Biophys. Acta Gen. Subj..

[33] Moskovitz J., Rahman M.A., Strassman J., Yancey S.O., Kushner S.R., Brot N., Weissbach H. (1995). Escherichia coli peptide methionine sulfoxide reductase gene: Regulation of expression and role in protecting against oxidative damage. J. Bacteriol..

[34] Rosen H., Klebanoff S.J., Wang Y., Brot N., Heinecke J.W., Fu X. (2009). Methionine oxidation contributes to bacterial killing by the myeloperoxidase system of neutrophils. Proc. Natl. Acad. Sci. USA.

[35] Belaaouaj A.A., Kim K.S., Shapiro S.D. (2000). Degradation of Outer Membrane Protein A in Escherichia coli Killing by Neutrophil Elastase. Science.

[36] López-Boado Y.S., Espinola M., Bahr S., Belaaouaj A. (2004). Neutrophil Serine Proteinases Cleave Bacterial Flagellin, Abrogating Its Host Response-Inducing Activity. J. Immunol..

[37] Fedorova M., Kuleva N., Hoffmann R. (2010). Identification of Cysteine, Methionine and Tryptophan Residues of Actin Oxidized In vivo during Oxidative Stress. J. Proteome Res..

[38] Abbenante G., Leung D., Bond T., Fairlie D.P. (2000). An efficient Fmoc strategy for the rapid synthesis of peptide para-nitroanilides. Lett. Pept. Sci..

[39] Morrison J. (1969). Kinetics of the reversible inhibition of enzyme-catalysed reactions by tight-binding inhibitors. Biochim. Biophys. Acta.

[40] Humphrey W., Dalke A., Schulten K. (1996). VMD: Visual molecular dynamics. J. Mol. Graph..

[41] Phillips J.C., Braun R., Wang W., Gumbart J., Tajkhorshid E., Villa E., Chipot C., Skeel R.D., Kalé L., Schulten K. (2005). Scalable molecular dynamics with NAMD. J. Comput. Chem..

[42] MacKerell A.D., Bashford D., Bellott M., Dunbrack R.L., Evanseck J.D., Field M.J., Fischer S., Gao J., Guo H., Ha S. (1998). All-Atom Empirical Potential for Molecular Modeling and Dynamics Studies of Proteins. J. Phys. Chem. B.

[43] De Veer S.J., Swedberg J.E., Akcan M., Rosengren K.J., Brattsand M., Craik D.J., Harris J.M. (2015). Engineered protease inhibitors based on sunflower trypsin inhibitor-1 (SFTI-1) provide insights into the role of sequence and conformation in Laskowski mechanism inhibition. Biochem. J..

