# Supplementary material for: Discrimination of Methionine Sulfoxide and Sulfone by Human Neutrophil Elastase
Source: Molecules. 2021 Sep 2;26(17):5344. doi: 10.3390/molecules26175344 (PMC8434204; doi:10.3390/molecules26175344)
Supplement: Supplementary file 1 [file molecules-26-05344-s001.zip › molecules-1339357-supplementary.pdf]

molecules

MDPI

Supplementary materials

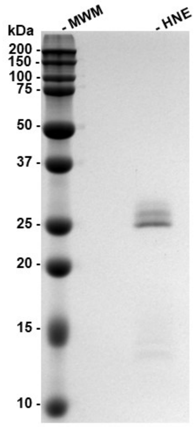

Figure S1. Trajectories for molecular dynamic simulation of HNE in complex with RMAV-H peptide aldehyde and its oxidized variants.

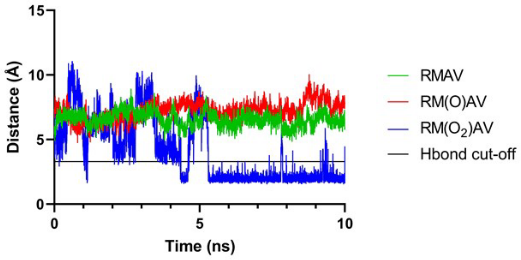

Figure S2. SDS PAGE analysis of HNE supplied by Elastin Products Company.

Table S1. Calculated and observed masses of candidate peptide-pNA substrates and peptide aldehyde inhibitors. All masses are listed in Daltons.

Substrate	Calc. Mass (M+1)	Obsv. Mass
Ac-RM(O_2)AV-pNA	670.29	670.3
Ac-RM(O_2)PA-pNA	668.27	668.3
Ac-RM(O_2)AA-pNA	642.26	642.2
YM(O_2)AV-pNA	635.24	635.3
Ac-REPA-pNA	634.28	634.3
RM(O_2)AV-pNA	628.28	628.3
RM(O_2)PA-pNA	626.26	626.3
YEAV-pNA	601.24	601.3
RM(O_2)AA-pNA	600.25	600.2
REPA-pNA	592.27	592.3
MeOSuc-AAPV-pNA	591.27	591.2
AM(O_2)AV-pNA	543.22	543.2
Ac-RM(O_2)AV-H	534.26	534.3
Ac-RM(O)AV-H	518.26	518.4
Ac-RMAV-H	502.26	502.3
